# The adaptive response to iron involves changes in energetic strategies in the pathogen *Candida albicans*

**DOI:** 10.1002/mbo3.970

**Published:** 2019-12-01

**Authors:** Celia Duval, Carole Macabiou, Camille Garcia, Emmanuel Lesuisse, Jean‐Michel Camadro, Françoise Auchère

**Affiliations:** ^1^ Laboratoire Mitochondries Métaux et Stress Oxydant Institut Jacques Monod UMR 7592 Université Paris‐Diderot/CNRS (USPC) Paris France; ^2^ Plateforme Protéomique structurale et fonctionnelle/Spectrométrie de masse Institut Jacques Monod UMR 7592 Université Paris‐Diderot/CNRS (USPC) Paris France

**Keywords:** *Candida albicans*, filamentation, iron, metabolism, mitochondria, oxidative stress

## Abstract

*Candida albicans* is an opportunist pathogen responsible for a large spectrum of infections, from superficial mycosis to systemic diseases known as candidiasis. Its ability to grow in different morphological forms, such as yeasts or filamentous hyphae, contributes to its survival in diverse microenvironments. Iron uptake has been associated with virulence, and *C. albicans* has developed elaborate strategies for acquiring iron from its host. In this work, we analyze the metabolic changes in response to changes in iron content in the growth medium and compare *C. albicans* adaptation to the presence or absence of iron. Functional and morphological studies, correlated to a quantitative proteomic analysis, were performed to assess the specific pathways underlying the response to iron, both in the yeast and filamentous forms. Overall, the results show that the adaptive response to iron is associated with a metabolic remodeling affecting the energetic pathways of the pathogen. This includes changes in the thiol‐dependent redox status, the activity of key mitochondrial enzymes and the respiratory chain. Iron deficiency stimulates bioenergetic pathways, whereas iron‐rich condition is associated with greater biosynthetic needs, particularly in filamentous forms. Moreover, we found that *C. albicans* yeast cells have an extraordinary capability to adapt to changes in environmental conditions.

## Graphical abstract

We analyze here the adaptive response of *Candida albicans* cells to changes in the iron content of the culture environment. This response includes changes in intracellular redox status and the reorientation of metabolic pathways, as shown by label‐free analyses and biochemical measurements. We found that iron deficiency stimulated the TCA cycle, mitochondrial respiratory chain, and ATP production, to compensate for cellular stress, to maintain normal levels of ATP, and to ensure cell survival. Conversely, an increase of iron is associated with biosynthetic needs, especially in filamentous forms.

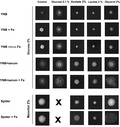

## 1 INTRODUCTION

*Candida albicans*, one of the predominant opportunist pathogen yeast, is a commensal organism that colonizes the mucosal surfaces of the oral and vaginal cavities and the digestive tract. *Candida albicans* infections extend from superficial mycosis to life‐threatening opportunistic bloodstream infections, which can develop into disseminated candidiasis, principally in patients with compromised immunity (Calderone, 2002; Mavor, Thewes, & Hube, 2005; Odds, 1988). A striking feature of *C. albicans* is its ability to grow in various morphological forms, including unicellular budding yeasts, filamentous pseudohyphae and true hyphae, and some less common forms, such as chlamydospores and opaque cells (Berman, 2006; Calderone, 2002; Gow, 1997; Sudbery, Gow, & Berman, 2004; Whiteway & Oberholzer, 2004). This ability to switch between forms is a key survival mechanism in the hostile host environment. Indeed, the hyphal form is invasive and can promote tissue penetration in the early stages of infection, whereas the yeast form may be more suitable for dissemination in the bloodstream (Calderone & Fonzi, 2001; Lo et al., 1997; Mitchell, 1998; Soll, 2002).

During infection in vivo, *Candida albicans* must adapt to host microenvironments with different key micronutrient and iron contents. For example, the pathogen faces extremely low free iron levels in the bloodstream during systemic infection, whereas it encounters much higher levels of free iron as a commensal within the mammalian gastrointestinal tract (Chen, Pande, French, Tuch, & Noble, 2011; Miret, Simpson, & McKie, 2003; Raymond, Dertz, & Kim, 2003). Host inflammatory responses to pathogens like *C. albicans* result in a further lowering of serum iron concentration, through mechanisms such as decrease in intestinal absorption and retention within reticuloendothelial cells (Prentice et al., 2012; Yang et al., 2002). Hepcidin plays a major role in lowering serum iron by inhibiting ferroportin, the mammalian iron‐export protein (Donovan et al., 2005; Nemeth et al., 2004). Therefore, when hepcidin levels are high, enterocyte absorption of dietary iron and release of macrophage iron to serum are blocked. For this reason, iron availability plays a crucial role in host‐pathogen relationship, determining whether the pathogen can acquire the necessary iron it requires while defending against iron toxicity. Furthermore, as iron is an essential element, its uptake is considered a virulence attribute and it has been suggested that colonization can occur only if the pathogen has access to sufficient iron (reviewed in (Sutak, Lesuisse, Tachezy, & Richardson, 2008)). For example, the treatment of endothelial cells with the iron chelator phenanthroline decreases damage due to *C. albicans* (Fratti, Belanger, Ghannoum, Edwards, & Filler, 1998). In addition, siderophore uptake by Sit1p/Arn1p is required in epithelial invasion and penetration (Heymann et al., 2002) and the high‐affinity iron permease is essential for *C. albicans* virulence (Ramanan & Wang, 2000). Moreover, it has recently been shown that limiting iron levels with the novel chelator DIBI (a hydroxypyridinone‐class chelator) inhibits *C. albicans* growth and increases susceptibility to azoles in a murine model of vaginitis (Savage et al., 2018). This may account for the greater susceptibility to *C. albicans* infection of patients suffering from diseases with symptoms of iron overload (Bullen, Rogers, Spalding, & Ward, 2006). Moreover, infection seems to disturb global iron homeostasis in the host in a murine model of candidiasis (Potrykus et al., 2013).

Iron is not readily available to pathogens in the human host, because it is mostly sequestered in association with specific proteins, such as the intracellular iron storage protein ferritin, which plays a key role in maintaining iron homeostasis or serum transferrin, which can bind and transport ferric iron. *Candida albicans* has developed three high‐affinity iron acquisition systems for exploiting iron within its host (for reviews see (Almeida, Wilson, & Hube, 2009; Fourie, Kuloyo, Mochochoko, Albertyn, & Pohl, 2018; Noble, 2013; Philpott, 2006; Sutak et al., 2008)). The first system is a reducing pathway located in the plasma membrane and responsible for iron acquisition from host ferritin and transferrin, and was originally described by Knight, Lesuisse, Stearman, Klausner, and Dancis (2002). This system involves two surface ferric reductases, which catalyze the reduction of ferric iron to the ferrous form (Hammacott, Williams, & Cashmore, 2000; Knight et al., 2002; Knight, Vilaire, Lesuisse, & Dancis, 2005; Ramanan & Wang, 2000; Yamada‐Okabe et al., 1996; Ziegler, Terzulli, Gaur, McCarthy, & Kosman, 2011). The ferrous form is then reoxidized by Fet3 to ferric iron while being transported into the cell by Ftr1 (Stearman, Yuan, Yamaguchi‐Iwai, Klausner, & Dancis, 1996). Another 13 homologous genes, encoding putative ferric reductases, have been identified in the *C. albicans* genome (Baek, Li, & Davis, 2008; Mamouei, Zeng, Wang, & Wang, 2017). *Candida albicans* can also use host ferritin under standard in vitro conditions or directly from host epithelial cells in culture, by making use of a specific cell‐surface adhesion protein, Als3 (Almeida et al., 2008). This unique iron exploitation strategy combines hypha formation, adhesion, and iron uptake. Proteins involved in iron acquisition are also differentially expressed to compensate for changes in iron availability. For example, the expression of the ferritin receptor Als3, increases during hyphal development, and the inhibition of hyphal development leads to lower levels of this receptor and a lower capacity for ferritin binding (Almeida et al., 2009; Sorgo, Brul, Koster, Koning, & Klis, 2013).

*Candida albicans* can also acquire iron from siderophores, low‐molecular weight high‐affinity iron‐binding molecules produced by other organisms (Andrews, Robinson, & Rodriguez‐Quinones, 2003; Haas, 2003; Heymann et al., 2002; Hu, Bai, Zheng, Wang, & Wang, 2002; Lan et al., 2004; Lesuisse, Knight, Camadro, & Dancis, 2002). This opportunistic iron uptake strategy may be important in ecological niches containing several different types of microorganisms. *Candida albicans* can also produce hemolytic factors and use hemoglobin and other heme proteins as a source of iron, probably through binding to erythrocytes (Kuznets et al., 2014; Luo, Samaranayake, & Yau, 2001; Manns, Mosser, & Buckley, 1994; Moors, Stull, Blank, Buckley, & Mosser, 1992; Nasser et al., 2016; Watanabe et al., 1999). Free hemoglobin is bound by a hemoglobin receptor, Rbt5, on the fungal cell surface, followed by endocytosis of Rbt5‐hemoglobin complexes and release of ferrous iron by the heme oxygenase Hmx1 (Knight et al., 2005; Pendrak, Chao, Yan, & Roberts, 2004; Weissman & Kornitzer, 2004; Weissman, Shemer, Conibear, & Kornitzer, 2008) which is upregulated in conditions of iron deprivation (Santos et al., 2003).

Iron uptake is also linked to infection stage. In the initial stage of infection, the gene expression profile is characterized by the overexpression of genes involved in iron acquisition, due to limited iron availability; by contrast, during later stages of infection, more iron is available due to extensive tissue damage (Blankenship & Mitchell, 2011; Chen & Noble, 2012; Chen et al., 2011; Lan et al., 2004; Linde, Wilson, Hube, & Guthke, 2010). *Candida albicans* protects itself against iron toxicity through its conserved transcription factor, Sfu1, which directly represses genes encoding iron uptake factors, including components of the hemoglobin uptake system, the reduction‐based iron uptake system, and the Sit1 siderophore transporter (Chen et al., 2011; Lan et al., 2004; Pelletier et al., 2007). Like many fungi, *C. albicans* also produces the Hap43 transcription factor, which is functional in conditions of iron limitation and facilitates iron acquisition through transcriptional repression of the repressor Sfu1 (Baek et al., 2008; Chen et al., 2011; Hsu, Yang, & Lan, 2011; Singh, Prasad, Sinha, Agarwal, & Natarajan, 2011). However, in *C. albicans*, iron regulation is also controlled by a third transcription factor, Sef1, which can upregulate *HAP43* expression, thereby indirectly controlling Sfu1 (Blankenship & Mitchell, 2011). For this reason, and because it promotes the expression of iron uptake and virulence genes, Sef1 plays a key role in iron regulation in this pathogen. In the bloodstream, where the concentration of free iron is low, *SEF1* and *HAP43* are expressed and functional, promoting the expression of iron uptake genes. Conversely, during growth in the gastrointestinal tract, Sfu1 represses iron uptake genes and Sef1, thereby also indirectly repressing *HAP43*, and promoting iron utilization. However, little is known about the effect of iron on the metabolic pathways of the pathogen.

The aim of our study is to analyze the metabolic changes of *C. albicans* in response to changes in iron content in the growth medium. We compared the adaptive response of *C. albicans* in iron‐deprived conditions, to analyze the capability of the organism to resist to extreme conditions, and in presence of 100 μM iron citrate, which can be considered an iron‐rich condition but within the limit of tolerance of the cells. A detailed analysis of the biochemical adaptive response to changes in iron levels was performed, and the findings of these functional studies were validated and correlated with a quantitative proteomic and clustering analysis of protein abundance.

Overall, our results suggest that iron induces a metabolic remodeling response and that *C. albicans* yeast cells can adapt to a large set of iron conditions. The metabolic changes induced by iron include changes in thiol‐dependent redox status, affecting intracellular glutathione levels and reduced thiol groups, but also changes in the levels of key enzymes involved in energetic metabolism, such as the enzymes of the TCA cycle, the glyoxylate shunt, the pentose phosphate pathway, and the mitochondrial respiratory chain. This study allows to characterize the specific metabolic pathways underlying the striking ability of the pathogen to adapt to various iron levels in the environment.

## 2 MATERIALS AND METHODS

### 2.1 Yeast strains, media, and growth conditions

The virulent *C. albicans* wild‐type strain SC5314 (Gillum et al., 1984) was routinely cultured at 30°C in minimal YNB (Difco yeast nitrogen base plus 2% D‐glucose). Hyphal growth was induced by culturing this strain in YNB medium supplemented with 10% fetal serum at 37°C or in semisynthetic Spider medium at 37°C (Sigma) (Hudson et al., 2004; Liu, Kohler, & Fink, 1994; Odds, 1988). Since YNB medium is more acidic than YNB + serum and spider media, the pH of the YNB medium was adjusted to pH 7. All cultures were carried out from an overnight culture in YNB medium, inoculated in the morning at OD = 0.1 in liquid medium, until reaching exponential phase (OD = 0.7, ~6 hr growth), with vigorous shaking on a rotary shaker. Solid media were supplemented with 2% Bacto agar (Difco). Iron‐rich conditions were achieved by adding 100 μM iron citrate to the media described above. Iron deficiency conditions were obtained by culturing the cells in synthetic YNB *minus* FeCl_3_ medium (Sunrise Science Products). When necessary, this iron‐depleted medium was supplemented with various concentrations of iron citrate.

### 2.2 Determination of iron content in the culture medium

Iron levels were determined by evaluating the colorimetric reaction of ferrous iron with bathophenanthroline disulfonic acid (BPS). In this assay, the various culture media were incubated with 0.1 M MES buffer (pH 4.5) and sodium dithionite, to reduce iron to the ferrous form for reaction with BPS. The addition of 5 mM BPS led to the formation of a pink complex absorbing at 535 nm. Iron content was then calculated by comparison with a standard curve generated with known concentrations of iron sulfate.

### 2.3 Measurement of iron uptake and ferric reductase activity

Iron uptake was measured in microtitration plates. Cells were grown overnight in YNB, and inoculated in the morning at OD = 0.1 in YNB, YNB + serum or Spider medium with or without iron citrate supplementation, and cultured for ~6 hr (at 30°C for the yeast forms and 37°C for the filamentous forms), following the protocol established in (Lesuisse, Raguzzi, & Crichton, 1987) showing that the optimal response to iron deficiency was obtained after 6 hr growth. They were then washed once with 2% EDTA, three times with distilled water and suspended in 50 mM citrate (trisodium) buffer (pH 6.5) containing 5% glucose. The cell suspension was dispensed into the wells of a microtiter plate, iron was added (as ^55^Fe (III) citrate) to a final concentration of 10 μM, and the plate was incubated for 15–60 min at 30°C. The cells were collected with a cell harvester (Brandel) and washed on the filter. All the uptake data presented in this study are expressed in picomoles of reduced iron/OD/hr.

For the measurement of ferric reductase activity, cell cultures were inoculated at an OD_600__nm_ = 0.1 from an overnight culture in YNB medium and cultured for 6 hr in the same media as described above. Cells were then collected by centrifugation for 5 min at 4,000 *g,* and the pellets were washed once with distilled water. The pellets were suspended in 1 ml of 20 mM citrate buffer (pH 6.5) supplemented with 5% glucose to obtain an OD_600__nm_ ~ 2, and the cell suspension was incubated in the dark in the presence of 1 mM ferric citrate and 5 mM BPS. Iron reduction by the cells was stopped by addition of 100 μl of 10 N HCl, and the samples were centrifuged for 3 min at 14,000 *g*. The absorbance of the pink complex was measured at 535 nm (*ε* = 19.5 mM^−1^ cm^−1^), and ferric reductase activity is expressed in nmoles of ferrous iron produced/OD/hr.

### 2.4 Preparation of *Candida albicans* cell extracts

*Candida albicans* cultures were inoculated at OD_600nm_ = 0.1 from an overnight culture in YNB medium. Cells were then cultured to an OD600nm of 0.7, corresponding to an early‐log phase, in the classic YNB, YNB + serum, and Spider media, and in the same media depleted in iron (for YNB) or supplemented with 100 μM iron citrate. Cells were then harvested by centrifugation. Filamentous forms were subjected to sonication before OD measurement, to disrupt the filaments. The pellets were resuspended in 50 mM potassium phosphate buffer pH 7.8 in the presence of protease inhibitors, and the cells were disrupted with glass beads and centrifuged for 30 min at 5,000 *g*. The supernatant was used as the crude cell extract.

### 2.5 Isolation of *Candida albicans* mitochondria

*Candida albicans* cells were cultured overnight in the appropriate medium and harvested by centrifugation at 4,000 *g* for 10 min at 4°C, washed with 50 ml of ice‐cold water and resuspended in 0.6 M sorbitol, 50 mM Tris‐HCl pH 7.5 supplemented with complete protease inhibitor mixture (Sigma). Cells were disrupted by vortexing with 0.45 mm‐diameter sterile glass beads six times, for 30 s each, at 2‐min intervals, on ice. All subsequent steps were carried out at 4°C. Glass beads and unbroken cells were removed by centrifugation at 4,000 *g* for 10 min, and the supernatants were centrifuged again (14,000 *g*, 10 min, 4°C). The resulting pellets (corresponding to the mitochondria) were resuspended in 0.6 M sorbitol, 50 mM Tris‐HCl, pH 7.5, and the supernatants constituted the cytosolic fraction. Purified mitochondria were immediately frozen at −80°C.

### 2.6 Determination of protein content

We used the Bradford protein assay, with bovine serum albumin as the standard, to determine the protein content of cell extracts and isolated mitochondria. Enzyme activities are expressed per milligram of protein.

### 2.7 Determination of glutathione levels

Glutathione levels were determined with a modified version of the Tietze recycling enzymatic assay, as previously described (Bulteau et al., 2012). For the estimation of total intracellular glutathione levels, samples were washed and resuspended in 50 mM potassium phosphate buffer pH 7.8 supplemented with ice‐cold 5% 5‐sulfosalicylic acid. Specific glutathione content was calculated from standard curves obtained with various concentrations of GSSG and is expressed in nmoles of glutathione/mg of protein. This assay is based on the reduction of each GSSG molecule to give two GSH molecules, and the reading is in GSSG equivalents because GSSG is used as the standard, so the total measured specific glutathione content is 0.5 GSH + GSSG. For the quantification of oxidized glutathione (GSSG), samples (including GSSG standards) were treated with 2% (vol/vol) 4‐vinylpyridine for 1 hr at room temperature before analysis. The cytosolic and mitochondrial GSH/GSSG ratios were calculated as follows: GSH/GSSG = 2 [(total glutathione)—GSSG]/GSSG.

### 2.8 Quantification of reduced thiol‐SH groups

Total reduced thiol content was determined by spectrophotometric quantification of the conversion of 5.5′‐dithiobis‐2‐nitrobenzoic acid (DTNB) into 5‐thio‐2‐nitrobenzoic acid (TNB) by the monitoring of absorbance at 412 nm (*ε* = 13.6 mM^−1^ cm^−1^) in cell extracts (total intracellular free thiol groups) (Sedlak, & Lindsay, 1968). Free thiol groups were determined, with the same protocol, in the supernatant after protein precipitation in TCA, and the values obtained are expressed in nmoles of reduced thiol/mg of protein.

### 2.9 Glucose‐6‐phosphate dehydrogenase activity

Glucose‐6‐phosphate dehydrogenase (G6PDH) activity was determined as previously described (Auchère et al., 2008), and the results are expressed in nmoles of NADPH/mg protein.

### 2.10 Citrate synthase activity

The citrate synthase activity of freshly prepared mitochondria was assayed in a reaction mixture containing 100 mM Tris‐HCl pH 8.0, 0.25 mM DTNB, 0.2 mM oxaloacetate, and 0.1 mM acetyl‐coA. The reaction was started by adding mitochondria, and the production of TNB through the reaction of free coenzyme A with DTNB was followed spectrophotometrically at 412 nm (*ε* = 13.6 mM^−1^ cm^−1^). The results are expressed in nmoles of TNB/min/mg protein.

### 2.11 Isocitrate lyase activity

Purified mitochondria were incubated in 100 mM potassium phosphate buffer pH 7.0, supplemented with 4 mM phenylhydrazine, 2.5 mM cysteine, and 2.5 mM MgCl2. The reaction was started by adding 2 mM isocitrate, and the production of glyoxylate phenylhydrazone was followed at 324 nm (*ε* = 19.3 mM^−1^ cm^−1^). The results are expressed in nmoles glyoxylate phenylhydrazone/min/mg protein.

### 2.12 Mitochondrial ATP titration

ATP was quantified in freshly purified mitochondria, with the Molecular Probes luminescence detection kit for ATP determination (Invitrogen). This assay is based on the requirement of ATP for light production by luciferase (emission maximum ~560 nm at pH 7.8) from the substrate luciferin. Freshly purified lysed mitochondria were added to the reaction mixture, which was shaken, and luminescence was then assessed immediately on a Spectramax microplate reader (Molecular Devices). ATP was quantified with a calibration curve established with various known concentrations of ATP. The results are expressed in picomoles ATP/mg protein.

### 2.13 *Candida albicans* cell viability and flow cytometry assay of ROS

Intracellular ROS production was detected by staining cells with the ROS‐sensitive fluorescent dyes DCFDA (2,7‐dichlorofluorescein diacetate, Sigma) or DHE (dihydroethidium, Sigma) and assessing staining with a FACScan flow cytometer (Becton Dickinson). Cells were cultured in the different media, centrifuged for 3 min at 4,000 *g*, washed and resuspended in PBS, and treated with 50 μM DCFDA for 30 min or 0.1 mg/ml DHE for 90 min in the dark. The cells were diluted, as appropriate, and fluorescence was measured immediately, only in living cells. Cell fluorescence in the absence of DCFDA or DHE was used to check that background fluorescence was similar in each strain, and the value obtained for untreated cells was subtracted from that obtained in each assay.

Cell viability was assessed by flow cytometry. Cells from an overnight culture in YNB medium were inoculated in the morning in the appropriate media at OD_600nm_ = 0.1 and grown for 6 hr, with and without iron supplementation (at 30°C for the yeast forms and 37°C for the filamentous forms). Cells were then centrifuged to remove the growth medium (3 min, 4,000 *g*). The pellets were suspended in 1 × PBS, and the samples were incubated for 30 min in the dark, in presence of 10 µg/ml propidium iodide. The cells were washed twice with PBS, and the samples were then diluted and analyzed by flow cytometry in microplates, with *λ*_exc_ = 488 nm and *λ*_em_ = 585 nm. The data were then analyzed with Cflow Sampler software. 2.17.

### 2.14 Quantitative analysis in label‐free experiments

For global proteomic analyses, the cells were grown from an overnight culture in YNB medium, inoculated at OD = 0.1 in the appropriate media and cultivated until reaching the early‐log phase (OD_600nm_ = 0.7) in presence of the appropriate iron content in the medium. The data of proteomic experiments are the results of three independent samples. Cell extracts were then prepared as described above, and the protein content of the samples was measured, in order to quantify the amount of proteins to be precipitated with acetone. Four times volume of cooled acetone (−20°C) were added to sample volume containing 50 µg of protein extracts. Vortexed tubes were incubated for 60 min at −20°C then centrifuged for 10 min at 13,000–15,000 *g*. The protein pellet was heated for 20 min at 95°C and then cooled on ice for 20 min before performing, in triplicate, digestion overnight at 37°C by sequencing grade trypsin (12.5 μg/ml; Promega, Madison, Wi, USA) in 30 μl of 25 mmol/L NH_4_HCO_3_. LC‐MS/MS acquisition was performed with a 2‐hr gradient. Label‐free quantification in between subject analysis was performed on raw data with Progenesis‐Qi software 4.1 (Nonlinear Dynamics Ltd, Newcastle, U.K.) using the following procedure: (a) chromatograms alignment, (b) peptide abundances normalization, (c) statistical analyses of features, and (d) peptides identification using the Mascot server through Proteome Discoverer 2.1 (Thermo Scientific). A decoy search was performed, and the significance threshold was fixed to 0.01. The resulting files were imported into Progenesis‐LC software. Normalized abundances of proteins from trypsin digests with similar normalized abundance variations (ANOVA *p* value < 0.05) were classified together by the AutoClass Bayesian clustering system (Achcar, Camadro, & Mestivier, 2009) and visualized with Javatreeview (http://jtreeview.sourceforge.net/).

### 2.15 LC‐MS/MS acquisition

All digests or protein extracts were analyzed using an Orbitrap Fusion Tribrid equipped with an EASY‐Spray nanoelectrospray ion source and coupled to an Easy nano‐LC Proxeon 1000 system (all devices are from Thermo Fisher Scientific, San Jose, CA). Chromatographic separation of peptides was performed with the following parameters: Acclaim PepMap100 C18 precolumn (2 cm, 75 μm i.d., 3 μm, 100 Å), Pepmap‐RSLC Proxeon C18 column (50 cm, 75 μm i.d., 2 μm, 100 Å), 300 nl/min flow, gradient rising from 95% solvent A (water, 0.1% formic acid) to 35% solvent B (100% acetonitrile and 0.1% formic acid) in 120 min followed by column regeneration for 50 min. Peptides were analyzed in the orbitrap in full ion scan mode at a resolution of 120,000 (at *m/z* 400) and with a mass range of *m/z* 350–1500. Peptides were analyzed in the Orbitrap cell, in full ion scan mode, at a resolution of 120,000 (at *m*/*z* 200), with a mass range of *m*/*z* 350–1550 and an AGC target of 4 × 10^5^. Fragments were obtained by higher‐energy C‐trap dissociation (HCD) activation with a collisional energy of 30%, and a quadrupole isolation window of 1.6 Da. MS/MS data were acquired in the ion trap in a top‐speed mode, with a total cycle of 3 s with an AGC target of 1 × 10^4^. The maximum ion accumulation times were set to 100 ms for MS acquisition and 35 ms for MS/MS acquisition in parallelization mode.

All MS/MS data were processed with an in‐house Mascot search server (Matrix Science, Boston, MA; version 2.4.1) using Proteome Discoverer 2.1 (Thermo Scientific). The mass tolerance was set to 6 ppm for precursor ions and 0.5 Da for fragments. The following modifications were used in variable modifications: oxidation (M), phosphorylation (STY), acetylation (Protein N‐term), deamidation (N, Q), HNE, Glutathione, and S‐Nitrosyl(C). The maximum number of missed cleavages by trypsin was limited to two for all proteases used. MS/MS data were searched against a in house *C. albicans* database built using *C. albicans* ORF database (*C_albicans*_SC5314_A21_current_orf_trans_all.fasta, 6,221 entries, 06–09‐2014) retrieved from the *Candida* Genome Database website (http://www.candidagenome.org/).

The mass spectrometry proteomics data have been deposited to the ProteomeXchange Consortium *via* the PRIDE partner repository with the dataset identifier PXD010881 (Vizcaino et al., 2016).

### 2.16 Statistical analysis

All data points in the figures, and the data provided in the tables are the means of at least three independent determinations. Student's *t* test or nonparametric Wilcoxon–Mann–Whitney tests were used to identify significant differences.

## 3 RESULTS

In *C. albicans*, the yeast form is favored by a temperature of 30°C and acidic pH (pH 4.0). Hyphal growth is promoted by a variety of environmental conditions such as a temperature of 37°C, the presence of serum, neutral or alkaline pH, 5% CO_2_ or 1 mM N‐acetylglucosamine (reviewed in (Sudbery et al., 2004)). In addition, hyphal growth is often induced in synthetic growth media, such as Lee's medium or the semisynthetic Spider medium (Lee, Buckley, & Campbell, 1975; Liu et al., 1994). In this study, we used two standard, robust and widely used experimental conditions to induce hypha formation in *C. albicans* SC5314 cells: the addition of serum and incubation at a temperature of 37°C and the semisynthetic Spider medium at 37°C (Hudson et al., 2004; Liu et al., 1994).

In order to compare the metabolic changes associated with changes in iron content in the growth medium, cells were grown until reaching the exponential phase in various conditions: The classic YNB, YNB + serum, and Spider media, and the same media depleted in iron (for YNB) or supplemented with 100 μM iron citrate. We used iron citrate because it is readily assimilated by the cells, does not modify the pH of the medium, and presents optimal bioavailability. We did not use iron chloride (FeCl_3_), because it precipitates at high concentrations in a pH‐dependent manner. In addition, since YNB medium is more acidic than YNB + serum and spider media, the pH of the YNB medium was adjusted to pH 7. Iron‐deficient conditions were achieved with synthetic YNB *minus* Fe medium. We decided to use this medium rather than iron chelators, such as bathophenanthroline (BPS), because this approach made it possible to control the amount of iron present in our culture media tightly. Furthermore, we have observed toxic effects of BPS in *C. albicans* cells and the presence of this molecule can disturb the analysis (data not shown). We measured the iron levels in our three culture media, as described in the experimental procedures. As expected, the minimal YNB medium contained 1.3 ± 0.1 μM iron. The presence of serum had no significant effect on iron concentration, which was 1.7 ± 0.2 μM in YNB + serum, and iron concentration was higher in the rich Spider medium, at 2.3 ± 0.3 μM. In YNB *minus* Fe medium, iron concentration was, as expected, below the detection limit of the assay. In this study, we decided to compare the adaptive response of the cells to iron‐deficient (YNB *minus* Fe) and 100 μM iron citrate, a condition representing a 100‐X excess compared to the YNB medium, but well within the usual limit of tolerance of *C. albicans* cells.

### 3.1 Influence of iron on *Candida albicans* morphology

**Figure 1:**
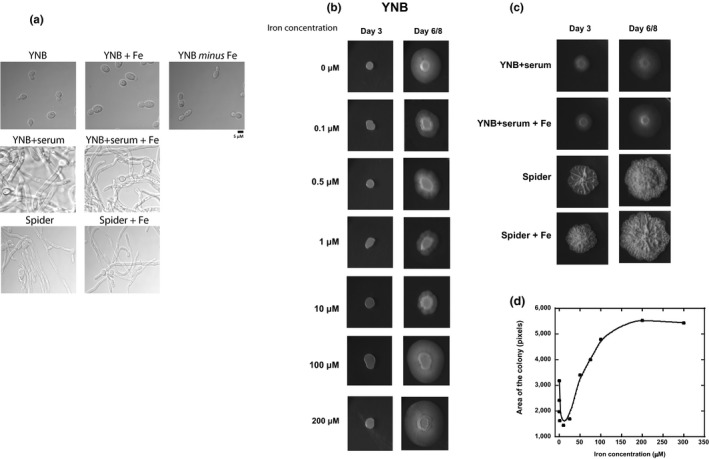
Effect of iron on the morphology of *Candida albicans* SC5314 cells grown on various solid culture media. (a) Morphology of *C. albicans* cells grown in liquid YNB, YNB + serum, and Spider media, supplemented or not with excess iron citrate, until reaching exponential phase. Cells were grown from an overnight culture in YNB medium, inoculated in the morning at OD_600__nm_ = 0.1, and grown until reaching OD_600__nm_ = 0.7 and images were taken with a contrast phase microscope (100X). Scale bar represents 5 μM. (b) Cells were grown in synthetic iron‐depleted YNB medium, supplemented with various concentrations of iron citrate. Plates were inoculated at an OD = 0.1 from an overnight culture and incubated for 6–8 days at 30°C. (c) Cells were grown in YNB, YNB + serum, and Spider media with or without supplementation with 100 μM iron citrate. Plates were inoculated at an OD = 0.1 from an overnight culture and incubated for 6–8 days at 30°C (YNB medium) or 37°C (YNB + serum and Spider media). (d) Areas of the colonies of panel B (measured with Image J software) as a function of iron concentration in the culture medium

Cells were cultivated in liquid and solid media, in presence or absence of iron citrate in the growth medium (Figure 1). For microscopic observations of Figure 1a, cells cultures were inoculated at OD = 0.1 (from an overnight culture in YNB medium) and cultivated until reaching exponential phase at OD = 0.7, exactly at the same time the cells were collected for all the biochemical assays presented in this study. The liquid culture assay tests the immediate ability of the organism to respond to environmental cues and initiate the growth of hyphae. Cells cultured in liquid YNB medium and inoculated at OD = 0.1 entered the exponential growth phase at the same time in the presence or absence of iron (data not shown). The microscopic analysis of *C. albicans* cultures grown to exponential phase (DO = 0.7) shows, as expected, a mixture of unicellular and budding yeast forms in minimal YNB medium at 30°C (Figure 1a). However, the cell morphology was not significantly modified by the presence or absence of iron. In YNB + serum and spider medium, germ‐tubes are usually visible after 30 min of growth. In Figure 1a,c*. albicans* cells observed in exponential phase grow in filamentous forms, a mixture of true hyphae with parallel‐sided walls, especially in YNB + serum, and pseudohyphae composed of branched elongated cells with constrictions at the septa. Indeed, the addition of serum and incubation at a temperature of 37°C has been described to generate a signal for the induction of hypha formation, and the Spider medium is supposed to induce more complex filamentous forms such as pseudohyphae (for a review see (Sudbery, 2011; Sudbery et al., 2004)). In this text, we will refer to pseudohyphae and hyphae together as “filamentous forms.” Based on the microscopic analysis of Figure 1a, the presence of 100 μM iron in the growth medium did not significantly affect the extent of filamentation of cells grown to exponential phase, whatever the conditions used to induce the filamentation process. However, similar morphological states could be associated to various intracellular status.

The solid medium assay tests the ability of the organism to develop yeast or hyphal growth in and around a colony depending of the availability of nutrients. *Candida albicans* cells were able to grow colonies in all our experimental conditions, on synthetic YNB *minus* Fe medium supplemented with various concentrations of iron citrate, from 0.1 to 300 μM (Figure 1b), and on YNB + serum and Spider media, with and without additional iron (Figure 1c). In YNB medium, yeast forms were observed for all the tested concentrations of iron, during the first five days of growth (Figure 1b). However, after 6/8 days of culture, cotton wool‐like filamentation similar to that observed in the presence of serum occurred, despite culture at 30°C, the temperature classically used to favor yeast forms. Filamentation levels were highest for the highest iron concentrations (100 and 200 μM). Surprisingly, *C. albicans* cells were also able to grow in conditions of iron deprivation (YNB *minus* Fe), in which a filamentation process was also observed after 6 days of culture. Furthermore, the area covered by the filamentous colony seems to be related to iron concentration, as shown on the bell curve in Figure 1d. Indeed, and quite surprisingly, the lowest levels of filamentation were observed in the presence of 10 μM iron. These results suggest that extreme iron conditions may trigger the yeast‐to‐hyphae transition as an adaptive response to the new growth conditions, and that iron concentration may influence the capacity of the pathogen to adapt its biomass.

On solid medium, filamentation in YNB + serum results in a “cotton wool”‐like appearance, whereas, in Spider medium, hypha formation leads to the development of feathery or spidery outgrowths from the main colony (Figure 1c). Interestingly, iron had no significant effect on the morphology of the filamentous forms (YNB + serum + Fe and Spider + Fe), and 100 μM iron even appeared to enhance the filamentation process in rich Spider medium (Figure 1c), suggesting that filamentous forms adapt more readily to such conditions.

### 3.2 Influence of iron on iron transport and ferric reductase activity of *Candida albicans* cells

**Figure 2:**
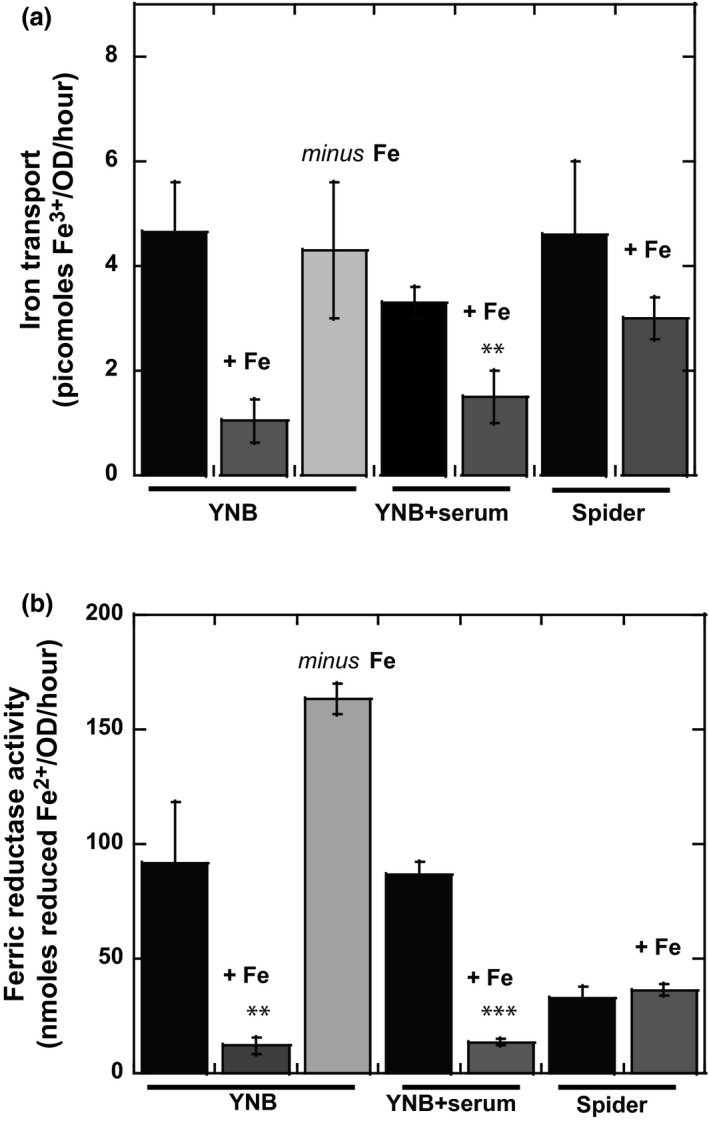
Effect of iron on iron transport and ferric reductase activity in *Candida albicans* SC5314 cells. Cells were grown in YNB, YNB + serum, and Spider media with and without supplementation with 100 μM iron citrate, or in a synthetic iron‐depleted medium (YNB *minus* Fe conditions). (a) Iron transport was measured in whole cells, with radiolabeled ferric citrate, as described in the experimental procedures; (b) Ferric reductase activity was assessed by monitoring the formation of a complex between ferrous iron (Fe^2+^) and BPS at 535 nm. All data points in the figure are the means ± *SD* of at least three determinations, normalized against the OD of the cell cultures, and Student's *t* tests were performed to identify significant differences (***p < .001 vs. control, **p < .01 vs. control)

Before assessing the potential metabolic changes associated with changes in the iron content of the culture medium, we had to evaluate the ability of *C. albicans* to take iron up under our experimental conditions. In vitro*, C. albicans* takes up iron *via* the reducing pathway located in the plasma membrane. The ferric reductases catalyze the reduction of ferric iron to the ferrous form, which is then transported into the cell by a ferrous transporter complex (Knight et al., 2005; Ziegler et al., 2011). Iron transport was assessed by following the incorporation of radioactive ^55^Fe (III) iron citrate into *C. albicans* cells cultured in various growth media, with or without iron supplementation, until reaching exponential growth phase. Figure 2a shows that cells grown in the presence of 100 μM iron citrate import less iron than those grown in iron‐depleted (YNB *minus* Fe) or in YNB or YNB + serum media, which contain around 1 μM iron. This was observed for both the yeast (YNB medium) and filamentous (YNB + serum and Spider) forms. Indeed, cells grown in the iron‐rich conditions probably have sufficiently high levels of iron and do not need to import as much iron when exposed to a new source of iron. In addition, these results suggest that the classic YNB medium is not an iron‐replete medium for *C. albicans*. However, iron transport levels in YNB + Fe were one quarter those in YNB, whereas iron uptake decreased by a factor of only 1.5–2 in the filamentous forms in the presence of 100 μM iron. These higher rates of iron import in the filamentous cells would suggest that these cells have higher requirements for continual iron import than do yeast cells. Interestingly, iron deficiency had no significant effect on the amount of iron imported into cells.

In parallel to iron uptake, we also measured ferric reductase activity, with significant effects observed for the yeast forms (Figure 2b). Indeed, enzymatic activity was greatly decreased by the culture of yeast cells in the presence of 100 μM iron, with a value of 12.1 ± 3.7 nmoles Fe^2+^/OD/hr in YNB + Fe, versus 91.9 ± 26.5 nmoles Fe^2+^/OD/hr in YNB medium. By contrast, ferric reductase activity was significantly higher, increasing to up to 163.5 ± 6.7 nmoles Fe^2+^/OD/hr, in the synthetic iron‐depleted medium (YNB *minus* Fe). This was expected because the *FRE10* gene encoding the major cell‐surface ferric reductase in *C. albicans* has been reported to be induced in conditions of low iron availability (Knight et al., 2005). However, these data indicate that yeast cells can rapidly regulate iron reduction at the plasma membrane to control iron uptake in response to changes in the iron content of the environment. Iron‐rich conditions also significantly decreased ferric reductase activity following the induction of filamentation in the presence of serum, with specific activities of 86.5 ± 5.6 nmoles Fe^2+^/OD/hr in YNB + serum and 13.6 ± 1.5 nmoles Fe^2+^/OD/hr in YNB + serum + Fe. A lower ferric reductase activity was found in Spider medium, which could be due to the higher level of iron in this medium (Figure 2b). However, the presence of iron did not affect ferric reductase activity if the yeast‐to‐hyphae transition was induced in Spider medium.

### 3.3 Influence of iron on intracellular glutathione levels and general thiol metabolism

**Figure 3:**
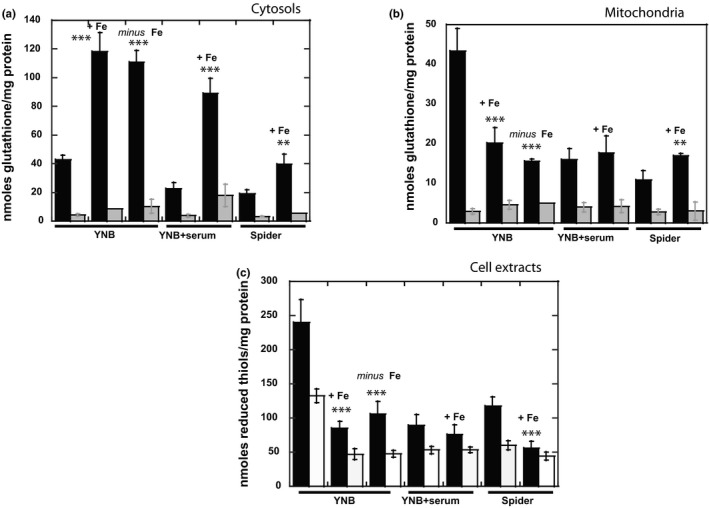
Effect of iron on intracellular glutathione levels and reduced thiol groups in *Candida albicans* cells grown in various culture media. Cells were cultured in YNB medium, YNB medium supplemented with 10% fetal bovine serum or Spider medium, until reaching exponential phase, with or without supplementation with iron excess, and glutathione levels were determined as described in the materials and methods section, in (a) the cytosol and (b) isolated mitochondria. Total glutathione levels are shown in black, and oxidized glutathione disulfide (GSSG) levels are shown in gray. (c) Reduced free thiol‐SH groups were determined in cell extracts (total reduced thiol groups are shown in black, free reduced thiol groups are shown in white). All data points in the figure are the means ± *SD* of at least three determinations, normalized with respect to the protein content of the samples, and Student's *t* test was used to identify significant differences (***p < .001 vs. control, **p < .01 vs. control)

We previously showed that the filamentation process is associated with a decrease in intracellular glutathione levels and a severe disruption of general intracellular thiol redox status (Guedouari et al., 2014). Several other studies, including work by our group on the yeast *S. cerevisiae*, have suggested a link between iron and thiol metabolism (Bulteau et al., 2012; Kumar et al., 2011; Rodriguez‐Manzaneque, Tamarit, Belli, Ros, & Herrero, 2002; Sipos et al., 2002). We therefore decided to explore the effect of iron on the intracellular thiol‐dependent redox status of the cells. Results of Figure 3 confirm a decrease of both cytosolic and mitochondrial total glutathione levels in filamentous forms, whatever the conditions used to induce the yeast‐to‐hyphae transition (YNB + serum or Spider medium). In addition, as previously reported, the mitochondrial GSH/GSSG ratio decreased, from 28 in YNB to 6 in YNB + serum or Spider medium, reflecting the occurrence of oxidative stress. Total cytosolic glutathione (GSH + GSSG) levels tripled, from 42.9 ± 2.9 nmoles/mg protein in YNB medium to 118.4 ± 13.2 nmoles in YNB + Fe medium, but the GSH/GSSG ratio remained unchanged at about 20 (Figure 3a). A similar effect was observed for the filamentous forms cultured in the presence of 100 μM iron, with total glutathione levels 4.5 times higher in YNB + serum + Fe than in YNB + serum, and only two times higher in Spider + Fe. However, no significance change was observed in the GSH/GSSG ratio, which remained at 10. These results indicate that, in response to an increase of iron in the growth medium, cytosolic glutathione levels increase in both the yeast and filamentous forms. In parallel, an increase of total glutathione was also higher in iron‐deficient conditions, at 110.8 ± 8.4 nmoles/mg protein. Both iron‐rich conditions and iron deficiency directly affected cytosolic glutathione levels.

Iron concentration had a strikingly different effect on mitochondrial glutathione levels (Figure 3b). Indeed, these levels decreased strongly in yeast forms, from 43.3 ± 5.8 nmoles/mg in YNB medium, to 20.1 ± 3.9 nmoles/mg protein in YNB + Fe, and 15.5 ± 0.6 in YNB *minus* Fe. However, the GSH/GSSG ratio was unchanged in YNB + Fe but decreased from 28 to 4 in YNB *minus* Fe, which reflects that the decrease of mitochondrial glutathione is associated with an increase of the oxidized GSSG form. Thus, the apparently normal growth of the cell in the absence of iron is associated with severe oxidative stress, probably the price to be paid for survival in these conditions, which may be associated with other functional disorders. By contrast, iron had no significant effect on the filamentous forms, in either YNB + serum or Spider medium. The presence of 100 μM iron in the growth medium affected cytosolic glutathione levels in both yeast and filamentous forms, but had consequences for mitochondrial glutathione levels only in yeast forms.

As previously reported, total reduced thiol group levels were much higher in yeast than in filamentous forms (Figure 3b). In YNB medium, the presence or absence of iron resulted in an important decrease of the number of reduced thiol groups, from 240 ± 33 nmoles/mg protein in YNB to 85.4 ± 9.8 and 106.2 ± 18.1 nmoles/mg protein in YNB + Fe and YNB *minus* Fe conditions, respectively. As total cytosolic glutathione levels increased under these conditions, these results suggest that other thiol groups may be affected. The levels of free reduced thiol groups decreased in both sets of conditions, suggesting a binding of thiol groups to proteins, by S‐thiolation, in response to changes in iron content.

### 3.4 Proteomic analyses and clustering

**Figure 4:**
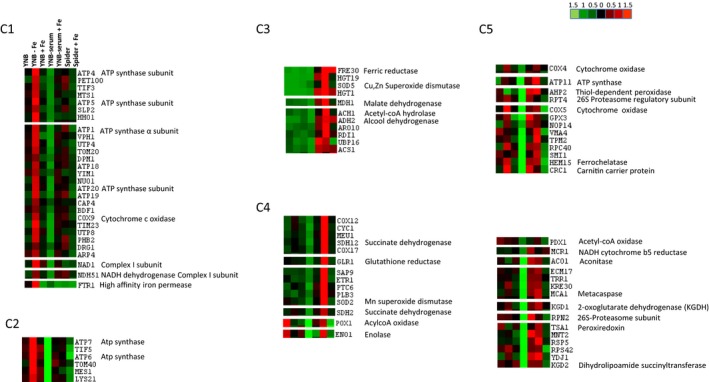
Classification of the proteins differentially expressed in *Candida albicans* cells cultured in YNB, YNB + serum, or Spider medium with or without iron citrate supplementation. Overview of JavaTreeView output from AutoClass clustering of proteins with different normalized abundances (ANOVA *p* value < .05). The data used for this classification were obtained in three separate biological experiments. The values are rendered in a red‐green color scale, where red represents higher expression and green indicates lower expression in the given experiment. Changes in protein levels are on a linear scale

In addition to morphological studies and thiol analysis, we performed a quantitative label‐free proteomic analysis, to decipher the specific metabolic pathways affected by changes in iron content in the growth medium. Cells were grown until reaching the exponential phase in various conditions: The classic YNB, YNB + serum, and Spider media, and the same media depleted in iron (for YNB) or supplemented with 100 μM iron citrate. Cell extracts were then submitted to mass spectrometry analyses, and the various abundances of the main proteins identified are presented in Table 1. The differentially expressed proteins were then classified with the Autoclass@IJM Bayesian clustering algorithm, in a specific cluster analysis of the label‐free data based on direct comparisons of protein abundance between the seven sets of conditions (Figure 4) (Achcar et al., 2009). This classification identified groups of proteins that could not be deduced directly from a classical visual examination of protein abundance profiles.

Proteins involved in iron metabolism were identified in the quantitative label‐free and clustering analysis shown in Table 1 and Figure 4. Indeed, the abundance of the high‐affinity iron permease Ftr1 was lower (by a factor of 10) in the presence of 100 μM iron, both in yeast and filamentous forms. The cell‐surface ferric reductase Fre30 was more abundant in YNB + serum + Fe conditions. However, the abundance of this protein was not affected by iron if filamentation was induced in Spider medium. In parallel, the abundance of the ferrochelatase protein was increased by culture in YNB + serum + Fe but was unaffected by culture in Spider + Fe.

When cocultured with a human oral epithelial cell line, invading *C. albicans* hyphae used a specific cell‐surface adhesin, Als3, to aggregate host ferritin on their surfaces (Almeida et al., 2008). Als3 also plays a key role in *C. albicans* adhesion to host epithelial and endothelial cells. Quantitative label‐free analyses showed Als3 abundance to be two to three times higher in the presence of 100 μM iron, in both yeast and filamentous forms (Table 1). Conversely, Als3 was less abundant in iron deficiency conditions (YNB *minus* Fe).

The results of the clustering analysis clearly reflected specific metabolic orientations according to iron concentration in the growth medium. For example, cluster C1 contained proteins that were more abundant in conditions of iron deficiency (YNB *minus* Fe). Cluster C1 contained various subunits of the ATP synthase complex and two components of the NADH dehydrogenase complex I of the mitochondrial respiratory chain. In addition, label‐free analysis (Table 1) showed the abundance of Cox 9, a subunit of complex IV cytochrome oxidase doubling in conditions of iron deficiency. Interestingly, some ATP synthase subunits were also present in cluster C2, which grouped together proteins more abundant in YNB *minus* Fe, and, to a lesser extent, in YNB + Fe conditions. Both iron‐rich conditions and iron deficiency stimulate the production of proteins involved in bioenergetics, but iron deficiency probably constitutes a more severe stress for the cells. Label‐free and clustering analyses of filamentous forms yielded more complex and unexpected results, with evidence for a different behavior of serum‐induced and Spider‐induced filamentous forms in presence of 100 μM iron. Indeed, we identified a set of proteins more abundant in YNB + serum + Fe than in YNB + serum (cluster C3), and a set of proteins less abundant in Spider + Fe than in Spider (cluster C4). Cluster C5 grouped together these two sets of conditions. Interestingly, the expression of specific proteins was induced, in presence of 100 μM iron, when the yeast‐to‐hyphae transition was induced by serum, but had the opposite effect when this transition was induced in rich Spider medium. In addition to ATP synthase and complexes of the respiratory chain subunits (complexes I and IV), the presence of 100 μM iron in YNB + serum led to an increase in abundance for almost all the enzymes of the TCA cycle, including citrate synthase, aconitase, succinate dehydrogenase, malate dehydrogenase, and some subunits of alpha‐ketoglutarate dehydrogenase (Table 1). Several proteins involved in defense against oxidative stress, such as the cytosolic superoxide dismutase, were also affected, together with several proteins involved in the maintenance of glutathione and thiol homeostasis, such as glutathione peroxidase Gpx2 and glutathione transferase Gtt1 (Table 1). The increase in glutathione reductase (Glr1) abundance helps to keep glutathione in its reduced state, as GSH, potentially counterbalancing the increase in mitochondrial GSSG (see Figure 3). The principal enzyme responsible for glutathione synthesis, gamma‐glutamylcysteine synthase (Gcs1), was also induced in YNB + serum + Fe. Proteins involved in fatty‐acid metabolism, such as fatty‐acid synthase subunits (Fas1‐2), and acyl and carnityl carrier proteins were also affected, suggesting that *C. albicans* cells may have additional needs for fatty‐acid synthesis to maintain cell survival in these conditions.

### 3.5 Influence of iron on the activity of specific enzymes involved in *Candida albicans* metabolic and energetic pathways

**Figure 5:**
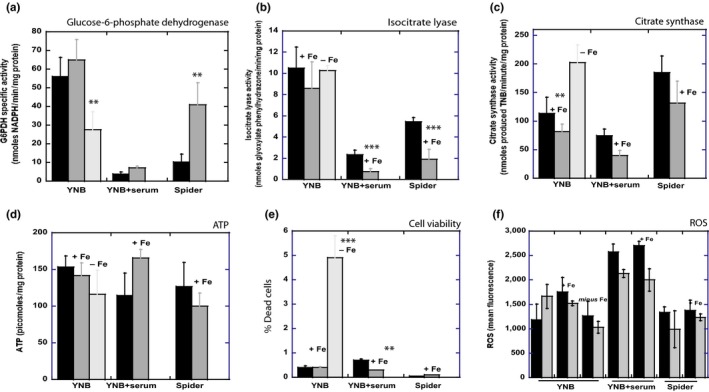
Effect of iron on specific activities of key enzymes, ATP production and cell viability in *Candida albicans* grown in different culture media. Cells were cultured in YNB medium, YNB medium supplemented with 10% fetal bovine serum or Spider medium, with or without iron supplementation. Enzyme activities were determined as described in the materials and methods, in whole‐cell extracts or isolated mitochondria. (a) Glucose‐6‐phosphate dehydrogenase activity, (b) isocitrate lyase activity, (c) citrate synthase activity. (d) ATP levels. (e) Measurement of % cell survival determined by flow cytometry, following cell labeling with propidium iodide. (f) Flow cytometry assay of ROS using the ROS‐sensitive fluorescent dyes DCFDA (black bars) or DHE (gray bars). All data points in the figure are the means of at least three determinations, normalized with respect to the protein content of the samples, and Student's *t* test was used to identify significant differences (***p < .001 vs. control, **p < .01 vs. control)

To validate the quantitative proteomic analysis, we focused on key enzymes involved in mitochondrial function and cellular energy metabolism. The changes in thiol redox status, including glutathione levels, observed in Figure 3, may affect the levels of intracellular NADPH, which provides most of the electrons required to maintain intracellular glutathione redox homeostasis. Glutathione reductases maintain the physiological GSH/GSSG balance by reducing GSSG in an NADPH‐dependent reaction. We previously showed that glucose‐6‐phosphate dehydrogenase, the first enzyme of the pentose phosphate pathway, was strongly inhibited in filamentous forms, indicating a shift in cell metabolism toward glycolytic and energetic pathways (Guedouari et al., 2014). This finding is consistent with the proteomic data in Table 1, showing a decrease in the abundance of this protein in filamentous forms. The presence of 100 μM iron had no significant effect on the G6PDH activity of yeast cells, but led to an increase in enzymatic activity in filamentous forms, with levels in YNB + serum + Fe twice those in YNB + serum, and a fourfold increase when filamentous forms were induced Spider medium (Figure 5a). Based on the data shown in Table 1, some of the increase in enzymatic activity observed in YNB + serum + Fe may be due to the fourfold increase in the abundance of the protein. Our data suggest that filamentous cells tend to shift toward synthetic pathways in the presence of iron, potentially facilitating adaptation of the cells to the new growth conditions. Interestingly, G6PDH activity was inhibited when yeast cells were placed in iron‐depleted growth medium, falling from 55.9 ± 10.3 nmoles NADPH/min/mg protein in YNB to 27.6 ± 19.5 nmoles NADPH/min/mg protein in YNB *minus* Fe (Figure 5a). In parallel, no significant change was observed in NADP‐dependent isocitrate dehydrogenase activity (cytosolic and mitochondrial), which may constitute an alternative pathway for NADPH production (data not shown). We explored the effect of filamentation on mitochondrial function further, by measuring the specific activities of two key mitochondrial enzymes: citrate synthase and isocitrate lyase (Figure 5b,c). Citrate synthase was strongly induced in yeast cells in conditions of iron deficiency (YNB *minus* Fe), but no parallel change in isocitrate lyase activity was observed, suggesting that iron deficiency has no effect on the glyoxylate shunt. In addition, cells grown in conditions of iron deficiency maintained almost normal intracellular ATP levels (Figure 5d). This would require stimulation of the TCA cycle and, thus, of citrate synthase, for the production of more ATP, because this enzyme catalyzes the reactions driving the TCA cycle within mitochondria. There is probably a price to pay for the ability to grow in such hostile conditions, as suggested by the ten times higher number of dead cells in YNB *minus* Fe than in the other conditions (Figure 5e). However, there was no significant effect of iron on the production of reactive oxygen species (superoxide anion and hydrogen peroxide) as shown in the flow cytometry measurements of Figure 5f. This indicates that the increase of dead cells observed in YNB *minus* Fe medium (Figure 5e) cannot be correlated to an excessive production of ROS. Inversely, the growth of cells in the presence of 100 μM iron led to lower levels of citrate synthase activity both in filamentous forms, and, to a lesser extent, in yeast forms (Figure 5c), suggesting that the cells displayed metabolic remodeling toward a less energetic pathway. These data are consistent with the increase in glucose‐6‐phosphate dehydrogenase activity observed in Figure 5a, reflecting the stimulation of pentose pathways and a shift toward synthesis and lower levels of energetic metabolism in cells. The glyoxylate cycle, which has several steps in common with the TCA cycle, allows this microorganism to use two‐carbon compounds as carbon sources and has been implicated in virulence (Lorenz & Fink, 2002). Interestingly, isocitrate lyase activity was inhibited in the presence of 100 μM iron, particularly in filamentous forms (Figure 5b).

## 4 DISCUSSION

Iron is an essential nutrient for all organisms and is involved in numerous biochemical processes, including cellular respiration, metabolism, and oxygen transport. It has also been suggested that the expression of virulence is linked to iron availability for pathogens such as *C. albicans* (Sutak et al., 2008). Indeed, the iron content of the environment is a key signal for initiating adaptation and facilitating access to host iron sources. It is therefore crucial to understand the metabolic changes associated with the response of *C. albicans* to changes in iron content of the growth medium and to identify some of the biochemical targets involved in this response. In this study, we decided to compare the adaptive response of *C. albicans* cells grown in iron‐deficient (YNB *minus* Fe) conditions and in presence of 100 μM iron citrate, a condition representing a 100‐fold increase compared to the YNB medium. We investigated the metabolic strategies used to deal with these different iron levels in the growth medium, by exploring changes in morphology, cellular redox status, enzyme activity, and protein abundance after induction of the filamentation process in vitro.

In *C. albicans*, hyphal growth is promoted by a variety of environmental conditions, and there are many ways to induce the filamentation process in vitro, including N‐glucosamine, low ammonium, or alkaline conditions (Sudbery et al., 2004). The signaling pathways transducing environmental signals into morphological switching are complex, and there is not a unique model of the filamentation conditions, very often resulting in confusing literature and discrepancies in the field. In this work, we have chosen two classic and widely used conditions to induce the filamentation process: The presence of serum at 37°C, which has been described to produce hyphae, and the semisynthetic Spider medium, which is supposed to induce more complex filamentous forms such as pseudohyphae (Hudson et al., 2004; Lee et al., 1975; Liu et al., 1994). Using two conditions to induce the filamentation may produce more complex results, but it emphasized on the complexity of *C. albicans* process of filamentation and adaptation. To control that the differences observed cannot be attributed to a medium effect, we systematically compared, for each medium, the control condition and the *minus* Fe or +Fe condition (for the yeast forms), and the control condition and the +Fe condition (for the filamentous forms). In addition, cells were cultivated strictly in the same conditions for all our experiments, including microscopic observations; therefore, Figure 1a shows the morphology of the cells at the time the cells were collected for further biochemical assays.

Microscopic observation of the cells grown to exponential phase shows a mixture of unicellular and budding yeast forms in minimal YNB medium at 30°C, and the presence of filamentous forms (a mixture of true hyphae and pseudohyphae) in YNB + serum and Spider media (Figure 1). Interestingly, *C. albicans* yeast cells were also able to grow in absence of iron. Moreover, the presence of 100 μM iron in the growth medium did not significantly affect the extent of filamentation. On solid medium, *C. albicans* cells grew and produced colonies at a very broad range of iron concentrations, from 0.1 to 300 μM iron citrate, corresponding to a 300‐fold increase over the iron levels in classic minimal YNB medium (Figure 1b). The cells even grew in an iron‐deficient synthetic medium (YNB *minus* Fe), in agreement with the microscopic observations of Figure 1a. This confirms that *C. albicans* turns on elaborate strategies to acquire iron sequestered by host iron‐binding proteins. However, the presence of 100 μM iron seemed to stimulate the yeast‐to‐hyphae transition, even in YNB medium, which favors the yeast form.

Similar morphology may reflect different intracellular metabolic status. Therefore, this ability to adapt to a large spectrum of iron concentrations in the environment must be associated with changes in cell metabolism, particularly in conditions of iron deficiency.

In Figure 2, iron uptake is lowered in the presence of 100 μM iron (Figure 2a), in both the yeast and filamentous forms, possibly because cells grown in such iron‐rich conditions already have sufficiently high levels of iron. Rapid changes in ferric reductase activity also facilitate the rapid regulation of iron reduction at the plasma membrane in the yeast forms (Figure 2b). Proteins involved in iron acquisition are also differentially expressed to compensate for changes in iron availability. In the filamentous forms, the ferric reductase reductive pathway may not be the main iron uptake system mobilized in our conditions. Indeed, expression of the ferritin receptor Als3 increases during hypha development, and the proteomic data of Table1 show an increase of the abundance of adhesin Als3 in the filamentous forms. *Candida albicans* cells may thus be able to obtain iron from the ferritin present in the YNB + serum and Spider media used to induce yeast‐to‐hyphae transition (Almeida et al., 2009).

These changes in iron uptake must be associated with specific metabolic changes to enable the cells to face new environmental challenges and to grow under all our experimental conditions. Several studies have suggested that there is a link between iron and thiol metabolism (Bulteau et al., 2012; Kumar et al., 2011; Rodriguez‐Manzaneque et al., 2002; Sipos et al., 2002). As previously reported by our group, filamentation was found to be associated with a decrease in total glutathione levels (Guedouari et al., 2014) (Figure 3). Moreover, cytosolic total glutathione levels changed significantly (~threefold) in response to changes in iron levels, in both the yeast and filamentous forms (Figure 3). Interestingly, cytosolic glutathione levels increase not only in response to 100 μM iron in the growth medium, but also in cells cultured in iron‐deficient conditions (YNB *minus* Fe). In parallel, total glutathione levels decreased in isolated mitochondria from the yeast forms in YNB + Fe and YNB *minus* Fe conditions. In addition, severe mitochondrial glutathione‐dependent oxidative stress was observed in YNB *minus* Fe conditions, with a lower GSH/GSSG ratio than in YNB medium (4 vs. 28). These data suggest that the intracellular glutathione pools are redistributed in response to iron in the yeast forms, with the import of mitochondrial glutathione into the cytosol, to help the cells to deal with the new conditions. However, these results clearly indicate that iron‐rich conditions induce an adaptation of the redox status of the cells, whereas iron deficiency leads to more severe conditions, including mitochondrial oxidative stress, with consequences for the subsequent adaptive response and survival of the cells. We also observed a filamentation process in yeast forms cultured in YNB + Fe (Figure 1), confirming the systematic association of filamentation with changes in glutathione content. Moreover, the decrease in free thiol groups (Figure 3b) suggests that thiol groups bind to the proteins via an S‐thiolation mechanism that may either protect the cells against oxidative damage or regulate intracellular thiol redox status.

**Figure 6:**
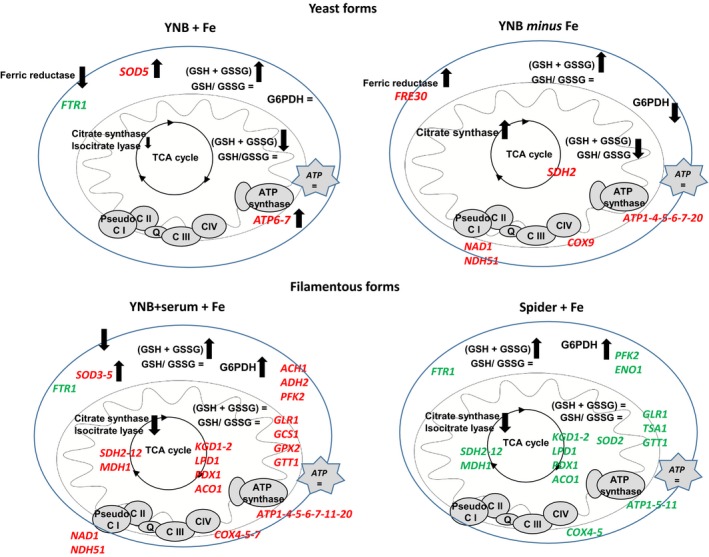
Schematic diagram of the adaptive response of yeast and filamentous *Candida albicans* cells to excess iron and iron deficiency. Changes indicate differences relative to control conditions (YNB + Fe vs. YNB) and the differences in protein abundance identified in proteomic and clustering analyses in Table 1 and Figure 4 are shown in italics

Interestingly, iron changes had no effect on the mitochondrial glutathione content of filamentous forms, whatever the conditions used to induce the yeast‐to‐hyphae transition (Figures 3 and 6). We hypothesize that cytosolic glutathione synthesis may occur de novo in filamentous forms in response to excess iron, possibly from intracellular γ‐glutamylcysteine stocks. This hypothesis is supported, at least for YNB + serum conditions, by the label‐free analysis, which revealed an increase in the abundance of the glutathione synthetase (Gcs1), but no increase synthesis of glutaredoxin Grx3/4 (Table 1). Another plausible explanation is that higher glutathione levels may result from reverse protein deglutathionylation, which can generate pools of free glutathione, or the import of glutathione from the rich Spider medium.

The global proteomic and clustering analyses (Table 1 and Figure 4), coupled to functional studies of specific enzymes involved in *C. albicans* metabolism, make it possible to characterize the specific metabolic pathways underlying the ability of the pathogen to adapt to changes in iron content in the growth medium. This metabolic remodeling is summarized in Figure 6, which illustrates the changes between classic conditions and iron‐rich or iron‐deficient conditions (YNB + Fe illustrates the differences between YNB and YNB + Fe). As already mentioned, all experiments were designed to eliminate major media effects, and for each medium, we kept the carbon sources and growth conditions strictly the same between experiments and the control conditions were systematically compared to the *minus* Fe or +Fe condition. As such, we were able, for example, to discriminate the difference due to the shift to an YNB to an YNB + serum or spider medium, from the effect of the addition of iron to the same media.

*Candida albicans* cells must occasionally face low iron concentrations within the host, but the YNB *minus* Fe conditions used in this study are particularly hostile and cannot be assimilated to iron‐limiting niches in the host. This condition is, however, of great interest because it emphasizes the extraordinary capability of the organism to resist to harsh conditions. *Candida albicans* cells were able to grow in such conditions, both in liquid and solid media (Figure 1), but at the expense of severe mitochondrial oxidative stress (Figure 3). Surprisingly, in *C. albicans* cells cultured in iron‐deficient conditions, we observe only 5% of cell death during the stationary phase, as shown in the cytometry assay of Figure 5e, and, on solid medium, cells were still able to develop after 6 days (Figure 1).

In YNB *minus* Fe, our data show a doubling of citrate synthase activity (Figure 5). Citrate synthase activation drives the TCA cycle toward more energetic metabolism, providing the mitochondrial respiratory chain with a larger number of electrons. Our findings are consistent with inhibition of the pentose phosphate pathway, reflected in the decrease in glucose‐6‐phosphate dehydrogenase activity, confirming the reorientation of cell metabolism toward bioenergetics pathways rather than biosynthesis. Our label‐free analysis also revealed an increase in the abundance of various mitochondrial respiratory chain complexes, such as complex I (Nad1, Ndh51), cytochrome oxidase (Cox 9), and various subunits of the ATP synthase complex. This strong stimulation of the bioenergetics pathway, coupled with an increase in iron uptake, allows the cells to deal with iron deficiency, to maintain normal ATP levels (Figure 5), and to survive glutathione‐dependent oxidative stress. This process does not seem to involve the glyoxylate cycle under our culture conditions, because levels of isocitrate lyase activity were unchanged. Regarding the response to iron deprivation, our data seem, in some aspects, in apparent contradiction with some previous publications. Indeed, the comparison of genome expression profiles published in (Lan et al., 2004) shows that several genes coding for proteins involved in TCA cycle (including citrate synthase) and electron transport chain are downregulated in “low iron” conditions. However, this paper also describes an overexpression of the gene coding for the high‐affinity permease Ftr1 in iron‐deficient conditions, as found in the proteomic analyses of Table 1. A Hap43‐dependent transcriptional repression of respiration was also observed under low iron conditions in (Chen & Noble, 2012). These apparent contradictory results could be explained by the use of different strains and media, since the plethora of media, strains, and in vitro conditions used to induce the filamentation process in the field of *C. albicans* often lead to discrepancies in the literature. Indeed, the strains used in references cited above are not the wild‐type SC5314 strain, and, most importantly, in the case of (Land et al. 2004), contains the mutation in IRO1, coding for a protein with a role in iron utilization. Furthermore, BPS was used to deprive iron and induce iron‐limiting conditions, very different from the iron‐deficient conditions induced by the synthetic YNB *minus* Fe medium. It is thus difficult to compare the metabolic adaptation of cells when grown in such contrasted conditions. In addition, our experiments were performed after growing the cells to exponential phase, and we cannot exclude that a prolonged iron restriction will give different results. Downregulation of TCA cycle and respiration has also been reported in *S. cerevisiae* in conditions of iron limitation (Philpott, Leidgens, & Frey, 2012; Puig, Askeland, & Thiele, 2005; Shakoury‐Elizeh et al., 2010). However, *C. albicans* presents an original metabolic response, with the presence of the enzymes of the glyoxylic shunt and a specific mitochondrial respiratory chain, comprising a real complex I (Li, She, & Calderone, 2016) instead of two NADH dehydrogenases in baker's yeast (Nde1 and Ndi1), a parallel respiratory chain (PAR) and a cyanide‐insensitive alternative oxidase. We have previously published that the filamentation process stimulates the alternative oxidase (Guedouari et al., 2014). For these reasons, we feel that it is not always relevant to compare data obtained with *C. albicans* with the ones regarding *S. cerevisiae*, especially when looking at metabolic responses.

In the presence of 100 μM iron, the metabolic response of *C. albicans* cells was different in yeast and filamentous forms. In the yeast forms, general cell metabolism did not change significantly, with only a slight decrease in citrate synthase activity. However, the abundance of some subunits of the ATP synthase complex increased, enabling the cells to maintain normal ATP levels in response to the new conditions (Figure 5d). When filamentous forms were cultured in the presence of iron, the TCA cycle was significantly inhibited, as shown by the lower levels of citrate synthase activity (~1.5‐fold lower) and inhibition of the glyoxylate shunt, with a significant decrease of isocitrate lyase activity (Figure 5). In parallel, the pentose phosphate pathway was strongly stimulated, in both YNB + serum and Spider medium. Thus, filamentous cell metabolism shifts toward biosynthesis pathways (consistently with the possible de novo synthesis of glutathione) and away from bioenergetic pathways in response to iron.

The scheme in Figure 6 shows that protein abundance also differed significantly according to the conditions used to induce the yeast‐to‐hyphae transition. The set of proteins induced in the YNB + serum + Fe conditions included ATP synthase and cytochrome oxidase subunits, and enzymes of the TCA cycle, such as α‐ketoglutarate dehydrogenase (Kgd1‐2), pyruvate dehydrogenase (Pdx1), and aconitase (Aco1). Overall, our results show that YNB + serum + Fe conditions are associated with a general induction of mitochondrial functions. Surprisingly, the abundances of a similar set of proteins decreased following the induction of filamentation in Spider medium (Figure 6). Similarly, opposite effects were observed for some proteins involved in glucose metabolism (Pfk2) or thiol metabolism (Glr1, Gtt1). These results suggest that, despite the similarity of the response to iron in terms of metabolic remodeling based on the functional activity of enzymes, the adaptive response may involve different biochemical mechanisms, depending on the conditions used to induce the yeast‐to‐hyphae transition. When filamentation is induced in Spider medium, the observed decrease in energetic metabolism is probably mostly due to changes in protein abundance, whereas this is not the case when filamentation is induced in the presence of serum. This may explain the minor (nonsignificant) differences in ATP levels between YNB + serum and Spider medium conditions (Figure 5d). A recent publication in the group of Jill Blankenship shows that gene expression in filamentous *C. albicans* can be different in solid or liquid medium and compares the differences existing between the various in vitro models both in the genetic requirements for filamentation and transcriptional responses to distinct filamentation‐inducing media (Azadmanesh, Gowen, Creger, Schafer, & Blankenship, 2017). As already mentioned, the signaling pathways of filamentation in *C. albicans* are rather complex and there is not a unique model of the filamentation conditions, very often resulting in confusing literature (Sudbery, 2011; Sudbery et al., 2004). A mutation that does not directly affect the hyphal growth process could nevertheless affect hyphal growth indirectly as a result of a change in any of the microenvironments. In our work, the choice of two classic conditions (serum and 37°C and spider medium) to induce the yeast‐to‐hyphae transition allowed to demonstrate that part of the adaptive response may be specific of the filamentous forms, but also emphasizes on the complexity of the process.

## 5 CONCLUSIONS

We analyze here the adaptive response of *C. albicans* cells to changes in the iron content of the culture environment. This response includes changes in intracellular redox status and the reorientation of metabolic pathways, as shown by label‐free analyses and biochemical measurements. We found that iron deficiency stimulated the TCA cycle, mitochondrial respiratory chain and ATP production, to compensate for cellular stress, and to maintain normal levels of ATP, to ensure cell survival. Conversely, an increase of iron is associated with biosynthetic needs, especially in filamentous forms. Overall, our results highlight the influence of the environment of *C. albicans* cells on the filamentation process and provide evidence for links between the effects of glucose and iron. In addition, our work provides evidence for differences between the yeast and filamentous forms, which seem to adapt more easily to new growth conditions. Overall, the observed metabolic changes allow the cells to maintain almost normal levels of ATP and cell viability in response to a wide range of iron conditions. This work provides new insight into the metabolic pathways involved in the original adaptive response of *C. albicans* to the broad range of iron conditions it encounters in the environment.

## CONFLICT OF INTEREST

None declared.

## AUTHOR CONTRIBUTIONS

Conceptualization, formal analysis, methodology, project administration, supervision, validation, and writing of original draft and revised version: F.A.; Conceptualization, formal analysis, methodology, and project administration: J.M.C.; Data curation and formal analysis: C.M. and C.D.

## ETHICS STATEMENT

None required

## Data Availability

The mass spectrometry proteomics data have been deposited to the ProteomeXchange Consortium *via* the PRIDE partner repository with the dataset identifier PXD010881.
